# A quarantine paradox: understanding Gender-Based Violence (GBV) in post-COVID-19 era: insights from Golden Valley mining community, Zimbabwe

**DOI:** 10.1186/s12889-024-20180-x

**Published:** 2024-10-01

**Authors:** Everjoy Magwegwe

**Affiliations:** South African Research Chair in South African Art and Visual Culture, University of Johannesburg, Johannesburg, South Africa

**Keywords:** Gender-based violence, COVID-19 pandemic, Survivor experiences, Community perceptions, Social-ecological model, Prevention strategies

## Abstract

The COVID-19 pandemic has brought unprecedented challenges globally, with a notable surge in gender-based violence (GBV) incidents. This descriptive, exploratory study conducted in the Golden Valley mining community in Kadoma, Mashonaland West Province, Zimbabwe, delves into the challenges faced by GBV survivors during and after the pandemic, alongside community perceptions of GBV in the post-COVID-19 era. Guided by Bronfenbrenner's Social-Ecological Model which offers insights into the various levels of influence on GBV behavior and experiences,aiding in the development of contextually relevant prevention strategies. The study used qualitative methods such as interviews and focus group discussions, there were 24 study participant classified by random and convenient sampling techniques including traditional and community leaders, gbv surviours medical personnel and male and female, community members. The research followed the COREQ guidelines to transparently document the research process and findings. The research sheds light on the multifaceted nature of GBV exacerbated by societal norms and economic instability in a low-income, masculine-dominated work culture experience in the COVID-19 period. Findings underscore the urgent need for comprehensive prevention programs, effective legislation, and community engagement to address GBV in post-pandemic communities. The research provides valuable lessons for enhancing GBV prevention efforts globally, emphasizing the significance of survivor voices and addressing root causes of GBV.

**Supplementary Information:**

The online version contains supplementary material available at 10.1186/s12889-024-20180-x.

## Introduction

The COVID-19 pandemic, which began in late 2019 and escalated globally in 2020, necessitated widespread quarantine measures that profoundly altered daily life and social interactions. Quarantine has been a critical public health strategy aimed at controlling the spread of infectious diseases. However, these measures, while essential for public health, paradoxically led to increased societal alienation and psychological distress among various populations, with impacts varying across demographics. The pandemic disrupted routines and exacerbated existing inequalities, leading to significant mental health challenges and financial insecurity across communities worldwide [49],Singh et al., 2021).

The psychological impact of the COVID-19 pandemic has been widely documented, with a considerable increase in anxiety and depression, particularly among vulnerable populations such as college students and low-income families [9, 22, 43]. While quarantine and lockdown measures were necessary for virus containment, they also heightened psychological suffering, including post-traumatic stress disorder, anxiety, and depression [17, 33]. Financial difficulties resulting from job losses and economic downturns further exacerbated these mental health disorders [4].

Moreover, the pandemic led to a significant increase in gender-based violence (GBV), often referred to as the "shadow pandemic" or the "quarantine paradox." GBV, already a severe issue affecting one in every three women worldwide, was exacerbated during the pandemic due to factors such as enforced house confinement, economic stress, and limited access to support services (UN Women 2021, [21],Yenilmez, 2020). The closure of schools and social services further trapped many women with their abusers, hindering progress towards gender equality [30].

During the COVID-19 pandemic, lockdowns caused significant setbacks for community-based prevention programs, forcing many groups to either suspend operations or transition to virtual platforms. While some successfully moved online, many struggled to adapt, especially those reliant on in-person community interaction. The transition was challenging due to planning issues, the limitations of remote platforms for certain initiatives, and funding constraints (Sileo et al., 2023). Additionally, many recipients faced barriers to access due to deficiencies in computer literacy. Despite the growing demand for GBV prevention programs, these efforts are underfunded and may overlap with other initiatives like mental health and HIV/AIDS [9]. Effective interventions require early, comprehensive, and multifaceted approaches, as well as enforced legislation, to achieve lasting change.

Post-pandemic, the long-term effects of increased rates of GBV are likely to persist. Research suggests that the psychological impact of violence during the pandemic could result in lasting mental health issues among survivors, including post-traumatic stress disorder (PTSD) and depression (Khanlou et al., 2021; [15]). Furthermore, the economic repercussions of the pandemic may deepen gender inequalities, making it harder for survivors to leave abusive situations [3]. As communities recover, it is crucial to prioritize interventions addressing the root causes of GBV and providing comprehensive support for survivors [5, 14].

In Zimbabwe, 59% of women aged 15–49 experienced physical and/or sexual violence by intimate partners. COVID-19 exacerbated the issue, making it more challenging for survivors to escape abuse (Croft et al., 2022). Despite laws against domestic violence, over 27% of women in unions reported GBV. The prevalence of violence rose significantly between 2015 and 2019. In 2019, emotional violence affected 22.1% of women, and physical violence affected 15.2% (ZIMSTAT & UNICEF, 2019). The Musasa Project reported 6,800 GBV cases in 2020, while the Ministry of Women Affairs documented incidents of economic abuse, physical assault, emotional abuse, and sexual harassment in 2021 [37],Ministry of Women Affairs, Community, Small and Medium Enterprises Development, 2021).

Despite the government's ongoing efforts to eradicate gender-based violence, these initiatives have been hampered by persistent macroeconomic instability and widespread job losses. The prevalence of gender-based violence has continued to rise, even with the widespread adoption of the women's empowerment mantra, underscoring the deep-rooted challenges in addressing this pervasive issue. While there is widespread recognition of the surge in GBV incidents during the COVID-19 pandemic, there remains a critical gap in understanding the nuanced challenges faced by GBV survivors, particularly in low-income, masculine-dominated communities such as mining communities in Zimbabwe. While existing research highlights the multifaceted nature of GBV and its exacerbation by societal norms and economic instability, there is limited insight into the post-pandemic landscape of GBV, including community perceptions, experiences, and policy implications. This research aims to fill that gap, providing valuable insights into post-COVID-19 community perceptions, experiences, and policy implications regarding GBV prevention. The study aims to offer lessons that can enhance prevention strategies in similar community setups worldwide.

This paper introduces gender-based violence (GBV) during COVID-19, research objectives, literature review, methodology, findings, and discussion. It explores post-pandemic community perceptions, experiences, and policy implications on GBV prevention. Data is collected from the Golden Valley mining community in Kadoma, Zimbabwe. The paper concludes with key insights, recommendations, and suggestions for future research.

### Aims and objectives of the research

The study aims to explore the impact of COVID-19 and lockdown measures on gender-based violence (GBV) within the Golden Valley mining community, examining the specific challenges that GBV survivors faced in the post-pandemic era. It will also investigate the community's perceptions and experiences regarding GBV prevention strategies, assess the policy implications of the evolving GBV landscape, and analyze how various factors within the social ecology influence GBV in this context.

## Research context

This study took place in Golden Valley, a mining community in Kadoma, Zimbabwe, in June 2022. The community is characterized by low income, inadequate infrastructure, and illegal mining activities. Adults in this community experience low levels of education, high rates of unemployment, deprivation, and limited access to essential services. Unemployment and deprivation exacerbate vulnerabilities, particularly for women. The prevalent mining culture in Golden Valley promotes harmful gender norms, leading to violence and discrimination against women. Male-dominated occupations, such as mining, reinforce aggressive behaviors towards women, hindering efforts to address gender-based violence (GBV). The COVID-19 pandemic has further exacerbated the situation, increasing incidents of violence against women. Gender norms and traditional beliefs that normalize violence against women continue to impede progress in addressing GBV. Golden Valley serves as a stark example of how socio-economic factors, cultural norms, and the pandemic intersect to perpetuate GBV. Addressing GBV in this context necessitates a deep understanding of these dynamics and the implementation of targeted interventions to create a safer community.

To gain a better understanding and address GBV in such contexts, Bronfenbrenner's ecological model provides a valuable framework. This model suggests that violence does not stem from a single cause but arises from multiple interacting influences at different levels of the social ecology, including individual, interpersonal, community, and societal levels. By applying this model, the study aims to shed light on how various social environments, from family and friends to broader societal norms, influence an individual's susceptibility to perpetrating or experiencing gender-based violence.

### The social-ecological model

**Fig. 1:**
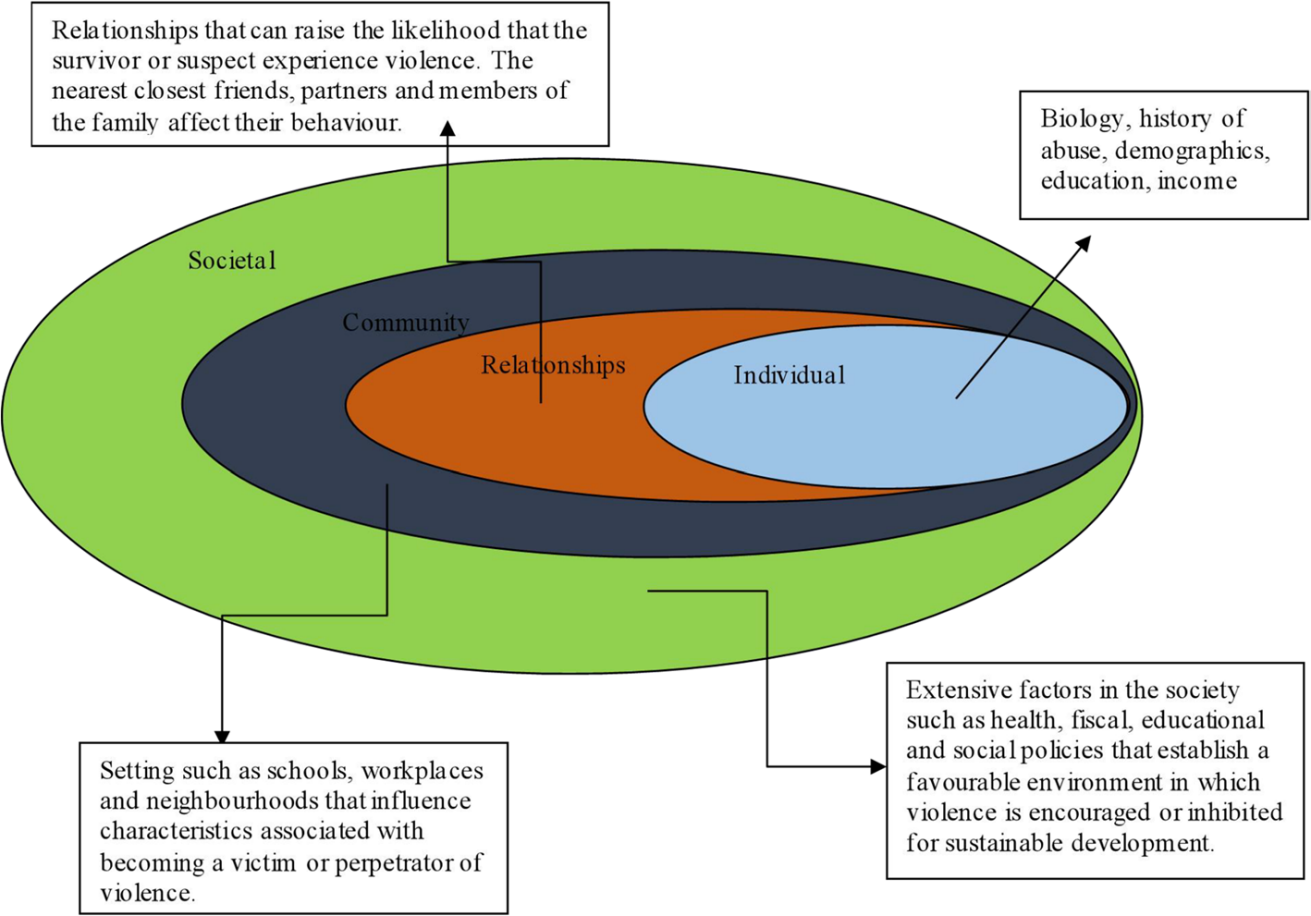
Violence risk factors and their interplay ecology Source: [19] with modification by author

Bronfenbrenner's ecological model, developed in 1979, examines human development by considering the individual and their environment. The model proposes that abuse does not result from a single factor but from multiple factors working together at various levels of the social ecology [46]. This framework is crucial for understanding GBV as it identifies risk factors and protective elements that can prevent individuals from becoming perpetrators or victims of GBV. The study applies this model, focusing on the societal aspects of gender-based violence. It emphasizes how an individual's social surroundings and relationships are influenced by their family, friends, schools, communities, and society at large. According to Kelly [24], violence does not have a singular cause but rather multiple levels of causation within the ecological framework. The diagram below illustrates how the model provides a framework for identifying risk factors and their interactions: (Fig. 1)

In the context of COVID-19, the model is significant in guiding the study, particularly at the individual level. It is noted that the stress, uncertainty, and worry brought on by the pandemic might exacerbate pre-existing mental health conditions or drug addiction, increasing the risk of gender-based violence (GBV) victimization or perpetration. Conversely, individuals with strong coping strategies and support systems may be better equipped to navigate and resist abusive situations [26, 27].

At the interpersonal level, it has been emphasized that lockdown-related restrictions may heighten power dynamics in close relationships, leading to increased coercion, control, and violence. Patterns of abuse could become more entrenched when individuals are isolated from external support systems. The epidemic has exacerbated gender norms and other social injustices, contributing to heightened tensions within communities as noted by Moreno et al. [35].

Moreover, the model illustrates that at the community level, financial strains within communities may worsen due to economic instability and job losses resulting from the pandemic. Financial pressures can lead to increased household tensions and the escalation of GBV, hindering individuals' ability to seek help or escape abusive situations due to strained or weakened community support networks. Additionally, the shift of institutions such as workplaces and schools to virtual spaces or the implementation of social distancing measures has altered contact dynamics and impacted individuals' access to resources for reporting abuse or seeking support [24]

## Research methodology

The research utilized a qualitative methodology to explore community perceptions, following the COREQ framework to ensure methodological rigor and transparency. The aim was to understand community perspectives on GBV during and post the COVID-19 pandemic. Qualitative methods like focus groups, interviews, and observations were employed for data collection. Adhering to COREQ principles guided the study's design, participant selection, data analysis, and result interpretation, enhancing the transparency and credibility of the study outcomes. Methodological rigor enhances the validity, reliability, and credibility of research findings, providing valuable insights into community viewpoints and experiences. A thematic analysis was used to analyze the data, focusing on recurring themes, patterns, and shared beliefs among participants.

### Population and sampling

The study involved 24 participants, with two Focus Group Discussions (FGDs) conducted, one with all female participants and the other with all male participants, randomly slected from a comprehensive list of eligible community members. Randomization was achieved through simple random sampling to ensure impartial and representative selection, thereby increasing the validity and generalizability of the study results. Other participant categories were selected through convenient sampling, with village chiefs, government officials, and church elders identified through purposive sampling. Additionally, the snowball sampling technique was used to identify survivors of sexual assault and victims of domestic abuse (Table 1).

**Table 1:** Summary of participants of the study

Category	Sampling Technique	Sample	Data Collection Instrument Used
Traditional Leaders	Purposive	2	In depth Interviews
Community leaders	Purposive	2	In-depth interviews
Survivors	Snowballing	3	Semi-structured interviews
Females members of the community	Random	8	Focus group discussions
Male members of the community	Random	8	Focus group discussions
Medical personnel	Convenient	1	In-depth interviews

1**. Summary of participants of the study**

### Data collection

In this study, methodological triangulation was employed through focus groups, key informant interviews, and participant observation to gather data. Data collection was facilitated using focus group discussion guidelines, interview guides, and observation guides(See Appendicies1,2,3 & 4). Interviewing traditional leaders, who serve as custodians of their communities' customs and heritage, aimed to unearth their profound knowledge of cultural history. Engaging with these leaders sought to reveal not only historical narratives but also the ongoing influence of cultural traditions on contemporary life, particularly in areas concerning governance, conflict resolution, and community unity. The insights provided by these leaders establish a vital connection between the past and present, underscoring the enduring importance of cultural heritage in shaping identity and social interactions.

Interacting with community leaders from mining communities was imperative as they possess profound insights into how mining impacts local livelihoods, encompassing economic, environmental, and social ramifications. These leaders often act as primary advocates for their communities, engaging in negotiations with mining companies and addressing issues like land disputes, displacement, and resource access. Their perspectives shed light on the real-world challenges and opportunities encountered by these communities, ensuring their voices are heard in discussions pertaining to mining and development. For the snowballing technique, a survivor identified through a community leader who intervened in a specific incident directed the researcher to other survivors for interviews. Focus group discussions for both male and female participants entailed similar questions to capture diverse viewpoints. Additionally, medical personnel were interviewed to provide insights from a health standpoint.

### Ethical consideration

This study was conducted in accordance with ethical guidelines established by the Institutional Review Board (IRB) at the University of Johannesburg. The research protocol was reviewed and approved by the IRB, ensuring that all procedures adhered to ethical standards and that the rights and welfare of participants were protected throughout the study. Informed consent was obtained from all participants, and measures were implemented to ensure confidentiality and minimize any potential risks.

The study protected participants' privacy and confidentiality by employing pseudonyms, securely storing data, and conducting talks in secret. Participants were advised of their rights and might withdraw at any moment. To reduce distress, delicate issues were handled with care, and participants were given the option of skipping questions or taking pauses as required. Referrals to local support services, such as counseling and helplines, were made in the event of difficulty, ensuring that participants had access to assistance if necessary.

### Limitations of the study

One of the study's key limitations was the difficulty of obtaining precise and trustworthy data on gender-based violence (GBV) in the Golden Valley mining community. GBV is frequently under reported due to cultural norms, shame, and fear of punishment, particularly in mining communities. The normalcy of violence, as well as the trauma endured by survivors, make comprehensive personal stories difficult to gather. Furthermore, the COVID-19 pandemic interrupted police and health services, resulting in gaps in records of GBV cases prior to, during, and post the pandemic. These limitations hinder the capacity to completely capture the real amount of GBV and its post pandemic effects.

Another significant constraint is the impact of COVID-19 on data collection and community involvement. Social distance and health problems limited possibilities for in-person interviews, focus group discussions, and direct observation, thereby limiting the data's depth. The study's findings may have limited generalizability since the Golden Valley mining community's distinct socioeconomic and cultural setting may not be representative of other mining communities in the country. Furthermore, restricted resources, such as time and finance, limited the breadth of the study, and possible researcher bias during observational methods may impact interpretations of community dynamics and behavior.

### Overview of the evidence

**T**he results of this study underscore the pervasive nature of GBV in the Golden Valley community during the COVID-19 pandemic, highlighting the intersection of socio-economic hardships, cultural norms, and strained social infrastructure. GBV, deeply rooted in unequal power dynamics and entrenched socio-cultural frameworks, was exacerbated by the pandemic, which heightened stress and economic pressures. The community’s isolation, lack of essential services, and normalization of violence further compounded the challenges faced by survivors, particularly women. Verbatim responses from participants reflect the lived realities of violence, illustrating how the pandemic intensified existing vulnerabilities and created new barriers to seeking help. Through focus group discussions and individual narratives, key forms of GBV physical abuse, sexual violence, and economic violence were identified as prevalent within households and the broader community, painting a complex picture of how COVID-19 worsened the GBV landscape in Golden Valley.

### Impact of lockdown on spousal dynamics and gender roles

For some participants, particularly those accustomed to the bustling world outside their homes, the initial days of confinement proved to be an unexpected source of strength and connection within families. However, as the lockdown persisted, a myriad of challenges emerged, shedding light on the intricate web of positive and negative impacts on relationships, especially within spousal dynamics. Many of the men who were questioned found it difficult to adjust to the ongoing lockdown and confinement in their homes. For other men, being trapped at home with their spouses was a trying situation and a cause of stress since it led to disagreements and conflict over little things in the house. It was unusual and frustrating for these men to spend their lockdown time at home with their wives, since they typically spend it at work or out with friends. Males began to notice their partners' shortcomings and incapacity to perform tasks during the lockdown, which suggested that males needed to exert control over women and caused tension in the household. relationships.

*"At first, the lockdown was actually kind of nice. We got to spend more time together as a family, and I enjoyed having my husband around more. But after a while, it started to get really stressful. We were all cooped up in this small house, and we started to get on each other's nerves.(Female, FGD).*

My husband, especially, had a hard time adjusting. He's used to being out and about, and he didn't like being stuck at home. We started arguing about little things, and it was really frustrating. I could tell that he was feeling trapped and stressed.

*It was a difficult time for us, but we managed to get through it. We learned a lot about each other and how to communicate better. And now that the lockdown is over, we appreciate the time we spent together even more."(Male,* **FGD).**

The results are in line with studies that demonstrate how confinement stress affected several areas and caused tension in the family's relationships.(Abu Hamid 2022, [39]) A few men, though, talked about how much they enjoyed having more time with their relationships.

The COVID-19 lockdowns had an influence on marital relations, emphasizing both challenges and opportunities for couples.Spending more time together heightened tensions and exacerbated pre-existing difficulties.Couples' tension and annoyance rose due to close quarters and a lack of social contacts, resulting in more intimate partner violence (IPV) episodes [32, 38]. Furthermore, lockdown examined couples' communication and conflict-resolution abilities. Effective communication and emotional intelligence are essential for resolving conflicts, especially during stressful situations.

Couples adapted to a new normal, developing strategies for dealing with conflicts that arose from living close together. Couples that used constructive conflict resolution strategies reported higher levels of relationship satisfaction [6, 12]. This emphasized the value of constructive dispute resolution. Positive home relationships, on the other hand, acted as a buffer against the stress of the epidemic, fostering resilience and emotional support. This contrast of experiences, in which some couples thrived and others struggled, demonstrates the intricacies of stressed-out relationships.

### Economic hardship, masculinity, and socioeconomic violence

The causes of socioeconomic violence are frequently linked to larger economic pressures and the household's general incapacity to satisfy its duties, rather than the acts or income loss of one gender [16]. When men fail to fulfill their obligations, socioeconomic violence might emerge (Kaplan, 2020). However, it is crucial to note that income generation is not primarily the duty of males, since women also make major contributions to home finances. The researcher observed that every respondent from every interview and discussion indicated this type of violence. For example, women explained how gender roles and duties were constructed in society, implying that males were considered to be the breadwinners who handled all money concerns and decision-making positions. One male respondent emphasized how their numerous frustrations were causing them to react violently to circumstances.

*The economic situation of the country as a whole has impacted negatively on our daily life and has caused most of us to act as if we have abandoned our responsibilities. Even before the onset of COVID-19 the economic situation* *was dire and it became worse during the lockdown as the economy just shutdown. Most men are into informal mining which does not translate to always having money* **(Male respondent, FGD).**

The concept of masculinity and what it meant to be a man was undermined by issues such as substance misuse, alcoholism, and unemployment, which resulted in a feeling of diminishing responsibility. The majority of the community residents had either experienced job loss, income reductions, or their means of subsistence had become unfeasible amid the lockdown. As a result, they had severe food insecurity and struggled to afford food. Families experienced stress, disagreements, and strife as a result of not having enough food. In their article "An Acute Crisis adds to Unresolved Chronic Crisis," Engel-Hills & Engel [16] draw the conclusion that the pandemic's acute crisis made the effects of the chronic crisis worse, which had a cumulative effect on families. They also note that pre-existing social issues may be seen as risks and pressures that have long been a part of communities' uneven social structures. Men claimed that women saw them as weak and worthless because they felt inadequate and unable to support their families, which made them feel less appreciated in both the community and the homes.

*"When men can't provide for their families, it causes a lot of problems. They get frustrated and take it out on us. It's like we're expected to be okay with it, just because he's the man of the house. It's not fair. The pandemic made things even worse. My husband lost his job, and we've been struggling to make ends meet. He's been drinking more and taking it out on me. I'm scared for my safety, but I don't know* where to turn." **(Female, FGD**).

The men were perplexed and angry because they couldn't come up with any plans or methods to positively showcase their masculinity. According to Conroy [11], when women challenge male dominance and break traditional gender roles, men become insecure and resort to violence as a form of resistance, violating patriarchal gender norms. The compensation hypothesis suggests that men lacking resources linked to being breadwinners use aggression to express frustrations towards women (Kaplan, 2020). A man shared his post-COVID-19 depression:

*It's tough being a man these days. I lost my job during the lockdown, and I feel like a failure. I can't provide for my family, and I'm starting to lose my mind. I know I'm taking it out on my wife, but I can't help it. I'm just so frustrated and stressed. I want to be a good husband and father, but I don't know how. I feel like I'm losing control of my life."(Male, FGDs).*

Respondents noted that the majority of physical abuse against spouses involved kicking, slapping, and whipping, especially prevalent when income came from mining activities. Economic dependence during the COVID-19 pandemic exacerbated the risk of abuse for many women, making it harder for them to leave abusive relationships. The lockdown and movement restrictions limited women's access to support services, exacerbating the situation. Mugisho [36] argues that economically dependent women are more likely to stay in abusive relationships. COVID-19 worsened this situation as many businesses closed, leaving women economically reliant on abusers. The lack of access to support services hindered women's ability to seek help or escape abusive situations due to financial constraints.

Traditional masculinity notions tie a man's worth to his ability to provide for his family. When men struggle to do so, they may experience feelings of worthlessness and anger, leading to violent behaviors. The compensation hypothesis suggests that men lacking resources may use aggression to assert masculinity and control. The interplay of masculine disregard, economic hardship, and gender norms can escalate into socioeconomic violence. The COVID-19 pandemic has emphasized the need for initiatives addressing economic empowerment for women and redefining masculinity in contemporary culture.

### Coping mechanisms

Alcohol is widely recognized as a catalyst for violence, weakening inhibitions, impairing judgment, and reducing the ability to recognize warning signs. Although alcohol may not directly cause gender-based violence (GBV), it was found to fuel such violence during the lockdown. Some men turned to alcohol and illegal activities to cope, exacerbating GBV cases and hindering response efforts. Increased alcohol sales and illegal alcohol consumption during the lockdown led to more abuse opportunities. Stress from financial challenges, family disputes, and workplace pressures drove men to alcohol, escalating domestic violence.

One respondent shared,

*"Alcohol's a big problem here. It's like a fuel for violence, especially when people get drunk. The lockdown made it worse. We need stricter laws and more support for those addicted. But it's not just about the laws; it's about how we see alcohol. We need to change that. It's not harmless; it's dangerous." (Male, FGD)*

*Another added,*

*"I've seen firsthand how alcohol can destroy families. Men come home drunk and start fights, and women end up getting hurt. It's a vicious cycle. We need to break it. We need to educate people about the dangers of alcohol, especially young people. We need to promote healthy alternatives. And we need to make it easier for people to get help if they're struggling with addiction." (Community leader,KII)*

These responses highlight the community's long-standing issue with alcoholism and its detrimental impact on family relationships during the epidemic. The normalization of alcohol consumption, combined with financial insecurity, isolation, and damaged family connections, created a dangerous environment where alcohol fueled gender-based violence. Men facing job loss and economic uncertainty turned to alcohol to cope, leading to increased hostility and violence at home. This escalation of GBV occurred in a vulnerable setting where alcohol consumption and pandemic-related stress heightened domestic violence.

The lockdown restrictions further confined many survivors with their abusers, reducing their ability to flee or seek help. Alcohol misuse aggravated the situation by compromising the abusers' judgment, increasing the frequency and severity of violence. The availability of illicit alcohol via unlawful stokvels facilitated access to drugs that fostered unhealthy habits. Furthermore, societal acceptance of alcohol and a lack of stringent rules during the epidemic hampered efforts to interrupt the cycle of violence. Calls for tighter restrictions and stronger support systems for people battling with addiction indicate an increasing recognition of alcohol's role in perpetuating GBV; yet, altering deeply established attitudes regarding alcohol usage remains a substantial problem.

Ultimately, the pandemic exposed the intersection between alcohol abuse and GBV in Golden Valley, revealing how societal stressors and substance abuse intertwined to create a heightened risk for women and families in a time of crisis.The COVID-19 lockdown has illuminated the critical relationship between alcohol abuse and gender-based violence. The stressors associated with the pandemic, combined with the normalization of alcohol consumption, have created a dangerous environment where violence is more likely to occur.

### Cultural acceptance and silence surrounding gender-based violence

According to the DHS of Zimbabwe (2022), women are physically abused by their husbands for extremely trivial causes. For instance, if a woman accidentally burns the meal she is preparing, her husband would have every right to beat her. One of the in-depth interview participants emphasized that a husband is entitled to discipline his wife in whatever manner he sees fit, and that this kind of behavior is accepted and socially acceptable in the community. Wife-beating is justified by African customs, where certain communities still maintain patriarchal systems that grant males authority over their spouses since wives are supposed to submit to their husbands at all times (Johnmary 2012). Because of this normalization, violence in the community has become systemic and ingrained. The traditional authorities and a survivor echoed this, saying that:

*Traditional practices accept the abuse of women (wife beating) for no valid reasons not to say that beating anyone is justified (unless it’s a law, for example, corporal punishment) In African culture, women are supposed to be submissive to their husbands or men in general from the community and if she fails to submit, it is deemed normal for the husband to punish her. Women accept this behavior from their husbands as they grow up being told that once they get married, they would be the property of the Husband. The coming of* *COVID did not change anything but rather made it worse for men to exercise more control in closed spaces (KII-Traditional leader).*

*"It's so frustrating. We're treated like property, like we don't have any rights. My husband beats me all the time, and there's nothing I can do about it. Everyone in the community knows what's happening, but they just look the other way. The pandemic was a nightmare. I was trapped with him, and there was no escape. I'm so tired of this. I just want to be free." (Female, Survivor)*

The aforementioned assertion demonstrates that the community views punishment as culturally appropriate. It is a private matter when abuse is acceptable and no one else in the community steps in to stop it. According to Ushe [47], the claim that women are socialized to be "silent" perpetuates gender inequality and leads to women's subjugation not just in the family but also in the community and across society. Reporting incidents become more difficult due to the already few resources available to access survivor-friendly centers.

*"You know, it's just the way things are around here. Men are the heads of the household, and women have to respect that. If they don't, well, there are consequences. It's been like that for generations. The pandemic made things worse. Women were stuck at home with their abusers, and there was nowhere for them to go. It's a sad situation, but it's the way it is. We need to focus on educating people about healthy relationships, but it's going to be a tough fight." (Male, FGD).*

Gender-based violence (GBV) is still strongly embedded in Golden Valley society in the post-COVID-19 era, making it extremely difficult for women to leave violent relationships. Traditional ideas continue to tolerate wife-beating as a kind of punishment for women who do not meet their husbands' expectations, a condition exacerbated by the epidemic, which increased men's authority and kept many women in harsh circumstances. The community's image of domestic violence as a private matter, along with patriarchal traditions that require women to submit, isolates women and inhibits their capacity to seek help. Despite greater awareness of the importance of good relationship education, altering these deeply held attitudes is a slow process. Community elders and traditional leaders highlight the importance of attitudes toward others in avoiding GBV, drawing parallels with social learning theories that claim that conduct is formed by contextual forces rather than being intrinsic. This viewpoint emphasizes the importance of human responsibility and proactive efforts, such as those used to combat COVID-19, in treating and preventing GBV.

One tactic recommended to deter GBV in the community was the employment of traditional courts. However according to one elder, the community is using traditional courts less frequently to resolve disputes of all kinds and prevent them from happening in the first place:

*Women and men would approach the traditional leaders with their grievances to have their disagreements resolved by the elders. The courts were respected and helped to ease problems in the community until COVID -19. Post COVID the community seem to have been forgotten. Perhaps the people have learnt to handle their own problems.* **(KII-Community leader)**

The aforementioned testimony highlights the significance of using indigenous methods to carry out activities for the development of communities. A respondent from a focus group discussion (FGD) said that traditional courts should be reinstated in the community despite their lack of use. This is because traditional courts include an Ubuntu element that promotes peace and unity both within families and the community as a whole.

#### Challenges and limitations in addressing gender-based violence

Participants noted an increased awareness of GBV as a pressing issue in the community following the pandemic. Public health crises highlighted the need for more comprehensive prevention strategies. Nonetheless, FGD members countered that despite the prevalence of GBV in the community, the police are overburdened with violent crimes linked to mining operations. Due to the mining operations, there are many violent criminals in the town, and the police receive daily reports of numerous attacks..

As demonstrated by the following testimony, insufficient institutional capacity resulted in a lack of follow-up and lengthy legal proceedings that deterred the reporting of GBV instances;

*We are supposed to educate the mining communities and neighboringcommunities to avoid GBV and to report cases when they occur; but we do* *little because of a lack of resources. Even the little we do is not continuous but only when we have facilitation from partners. Sometimes we do not have transport to do community sensitization or even to follow up on the case. As a result, people lose interest and abandon the case* (**KII -Police Official),**

This scarcity of resources not only impedes instructional initiatives, but it also inhibits law enforcement's capacity to respond effectively to recorded incidents. The lack of ongoing assistance and follow-up methods creates a climate in which victims may lose interest in seeking justice, continuing the cycle of violence (Cheng et al.,2022).

Difficulties in reporting GBV instances include community dysfunctional systems particularly in the police force. Even while services for justice, law, and order were offered, long-distance travelers could not easily reach them. It was mentioned that the amount of time it took to get justice, much alone for one to be served, proved to be a deterrent to seeking justice; A male responded from FGD stated:

*‘Even if you report, they ask for transport, you have to pay some money to open the file; you have to go to the police station many times…it is better if one did not report at all*’ **(FGD -Male group respondent).**

Survivors reported facing numerous challenges, including limited access to support services, and social isolation during lockdowns.

*People fight over mining claims, proceeds from the mining activities, and Others who are bullies in the industry. Hence if a victim of domestic violence report to the police they are told to go back home and try and work it out (FGD mixed group respondent).*

The police said that they were unable to handle every case that was reported due to the nature of business that takes place in and around the neighborhood, noting that some cases that required further investigation would simply be put on hold or even forgotten.

Following COVID-19, there has been an increase in community awareness of gender-based violence (GBV), but obstacles in addressing the issue persist. Police authorities, overburdened by violent crimes related to mining activities, frequently deprioritize GBV cases, leaving survivors without assistance. Despite national frameworks such as Zimbabwe's Domestic Violence Act, little resources for ongoing community education and institutional failings make reporting abuse difficult. Survivors confront challenges including economic insecurity and restricted access to assistance, which are exacerbated by the pandemic's constraints. GBV is widespread, with many survivors trapped in abusive settings.

#### A health perspective

The researcher's interactions with the medical staff revealed that they had received reports from women who had been abused by their husbands, and that the only things they could do for them were to treat them and occasionally provide counseling. However, these services were not widely available during the COVID-19 pandemic. Adding that to prevent a high death toll, all effort and little resources were directed at the containment of COVID-19.

*"I'll never forget the day a young woman came into our clinic with a black eye and bruises all over her arms. She was terrified, but she managed to tell me that her husband had beaten her. I treated her injuries and tried to offer her some comfort, but I knew that her pain went much deeper than her physical wounds.*

*It was heartbreaking to see how violence had shattered her life. She was afraid to go home, afraid to trust anyone. I referred her to a counseling service, but I knew that even if she received help, the scars of her abuse would stay with her forever”(Heath personel, KII).*

Despite the actors' best efforts, survivors' overall healthcare access remained limited owing to a shortage of funding. Some areas did not provide free services. In public waiting spaces, persons were forced to declare the reason for seeking care. The lack of anonymity caused GBV sufferers to shun services Nesset et al. [38]. Increased outreach is required for preventative programs to provide fair access to campaign information.

#### A religious perspective

Religious leaders were recognized as intermediaries in the prevention of GBV, to whom individuals occasionally turned for help, particularly with domestic family matters. The majority religion in the community is Christianity, while there are many other faiths as well. A church elder clarified in an interview that the church was a haven of peace and that people came to it for counseling, forgiveness, and general dispute settlement. The response emphasized that during COVID-19, the church conducted virtual services and used social media platforms like Whats App to interact with its members. In some ways, this helped. These platforms are still utilized in conjunction with physical platforms in the post-COVID-19 era. According to a female respondent from the focus group discussions, the church has functioned as a center of safety, providing a safe place for people to retreat to as tensions escalated.

*"As religious leaders, we frequently serve as intermediates in avoiding GBV, particularly in family situations. During COVID-19, we used virtual services like Whats App to keep connected, which was beneficial. Now we're combining this with physical gatherings. A woman in our focus group stated that the church become a safe haven during times of conflict."(Religious Leader, KII)*

The results of the study clarified that some GBV preventive techniques had been applied in the neighborhood. However, there is still a lack of clarity on the techniques and players involved in raising awareness among those addressing GBV in the community [23]. There is a need for more solid measures to be put in place to minimize occurrences of violence in the Golden Valley mining community because the services for GBV there are extremely inadequate, unsystematic, and intermittent in comparison to the major town of Kadoma. Programs aimed at preventing GBV in the Golden Valley mining community must address gender norms and expectations related to what is considered normal for both men and women.

#### Post-COVID-19 impact on GBV in golden valley

The post-COVID-19 scenario in the Golden Valley neighborhood, like many other disadvantaged areas, demonstrates the pandemic's long-term consequences on gender-based violence (GBV) and economic instability. While the acute crisis of the epidemic has passed, the aftermath has left long-term issues, particularly for women and disadvantaged populations. Economic recovery is gradual, with many men still struggling to find secure employment, particularly in informal industries like mining. According to one male respondent,

*“Even before the epidemic, the economic situation was poor, and it became worse during the lockdown" (Male, FGD).*

The epidemic increased pre-existing socioeconomic stresses, trapping many families in perpetual poverty, fueling stress, resentment, and violence. The post-COVID-19 atmosphere has made women more vulnerable, since economic dependency on their boyfriends endures, making it impossible to leave violent situations. Many women, particularly those who lost their careers during the epidemic, continue to lack a secure income or access to other employment opportunities [11]. Furthermore, this economic uncertainty, along with established patriarchal norms, has reinforced conventional gender roles, with men perceived as providers and women as dependents. When males are unable to fulfill these duties, they frequently turn to violence to vent their frustrations, resulting in more domestic abuse [23].

Access to support services remains restricted in the post-COVID era, with many critical services such as shelters, legal help, and counseling being underfunded or impossible to get in rural locations such as Golden Valley. The epidemic interrupted these programs, and complete recovery has been gradual, leaving many women without the means to seek assistance. As one female respondent stated,

*"I'm scared for my safety, but I don't know where to turn" (Female, FGD).*

The absence of institutional assistance and continuing economic difficulties have contributed to a long-term problem for GBV survivors.

Furthermore, social views regarding GBV have not changed greatly, and many detrimental conventions endure. According to one participant,

*“Some members of the community still think that men have the right to reprimand their wives, reflecting the group's long-standing support of violence”(Female, FGD)*

The compensating theory proposes that males, feeling emasculated by their incapacity to give, use violence to reestablish power (Kaplan, 2020). This dynamic continues to influence the post-pandemic world, making GBV a recurrent concern. The post-COVID-19 scenario in Golden Valley is marked by delayed economic recovery, women's ongoing economic reliance on their abusers, and pervasive socio-cultural norms that allow GBV. Addressing these difficulties needs long-term initiatives focused on economic empowerment, increased access to support services, and cultural change to combat negative gender norms.

## Conclusion

The post-COVID-19 scenario in Golden Valley has highlighted the long-term economic and social implications of GBV, particularly for women in undeserved regions. Economic insecurity continues to disproportionately afflict women, exacerbating their dependency on abusive spouses. Patriarchal standards and conventional gender roles persist, contributing to recurrent domestic violence. Furthermore, the epidemic has hampered access to key support services such as shelters, legal help, and therapy, leaving survivors without basic necessities. Although the acute phase of the pandemic has passed, the aftermath continues to affect the community's socioeconomic and gender dynamics, sustaining vulnerabilities that worsen GBV. Addressing these issues needs long-term, multifaceted efforts to interrupt the cycle of violence and help survivors.

Economic empowerment initiatives aiming to improve women's financial independence, particularly in Golden Valley, are recommended as solutions to these concerns. Initiatives that focus on job creation and financial literacy training might reduce women's economic dependency on abusive spouses. Cultural changes including intentional interaction with community and religious leaders in order to question and alter destructive patriarchal behaviors that perpetuate GBV are also necessary. Furthermore, peaceful forms of masculinity must be encouraged. To build a more integrated and effective response to GBV, key players (government agencies, non-governmental organizations, healthcare professionals, and law enforcement) must work together more closely.

Future research should investigate the long-term economic recovery of disadvantaged people after COVID-19 and how it relates to GBV rates. Further research is needed to determine the role of cultural and religious leaders in shifting gender norms and how such developments affect GBV prevention methods. Finally, an evaluation of the efficacy of existing GBV support services in rural areas is required, as is the creation of measures to increase access and quality of these programs.

## Supplementary Information

Supplementary Material 1.

## Data Availability

Not applicable
