# Supplementary material for: A quarantine paradox: understanding Gender-Based Violence (GBV) in post-COVID-19 era: insights from Golden Valley mining community, Zimbabwe
Source: BMC Public Health. 2024 Oct 1;24:2679. doi: 10.1186/s12889-024-20180-x (PMC11443934; doi:10.1186/s12889-024-20180-x)
Supplement: Supplementary file 1 — Supplementary Material 1. [file 12889_2024_20180_MOESM1_ESM.docx]

# Appendix 1: Women and Men Focus Group Discussion Guide

**Women and Men Focus Group Discussion Guide**

**Introduction:**

My name is Everjoy Magwegwe, and I am a Postdoctoral Research Fellow at the University of Johannesburg in South Africa. I am doing research on the influence of COVID-19 and lockdown measures on gender-based violence (GBV) in mining communities, with a particular focus on the Golden Valley community. The study intends to investigate the influence of COVID-19 and lockdown measures on gender-based violence (GBV) in the Golden Valley mining community, focusing on the unique issues that GBV survivors experienced in the post-pandemic era. It will also look at the community's opinions and experiences with GBV prevention efforts, assess the policy implications of the changing GBV environment, and examine how different components in the social ecology influence GBV in this community. I would like to ask you some questions about GBV in your community to better understand the situation and find ways to address it effectively.

If you feel uneasy, leave at any time. Participation in the discussion is entirely optional, and you are under no obligation to answer any questions you do not choose to. I have nothing to contribute other than listening; there will be no further direct benefits. I do not want your names, and I do not want to write them down. As a result, I will not share any additional identifying information in any content I make based on this issue. I will politely accept anything you say today, and we will only post your comments as general responses with those of all the others that speak with me. I also ask that you keep everything discreet. I will be recording this discussion to make sure that I do not miss what you have to say. I hope that this is OK with you? I want you to answer my questions however you want. There is no wrong answer to any question.

Have you have any questions before we start?

1. How has the COVID-19 epidemic affected your daily routines and community activities?
2. What additional obstacles or issues have you encountered in this community after the pandemic?
3. Have there been any cases of violence against men and women in your neighborhood since the pandemic?
4. Who do you believe are the most prevalent perpetrators of violence in your community, especially since COVID-19?
5. Without naming anybody, what kind of violence are women facing following the epidemic, and why do you believe this occurs?

6. What additional types of gender-based violence (GBV) have formed or increased in the community after COVID-19?
7. Does the epidemic affect the likelihood of survivors of violence reporting their experiences to others? Why, or why not?
8. How has COVID-19 changed the community's support systems and services for GBV survivors?
9. Have any new community structures or initiatives been established to support GBV survivors after the pandemic?
10. How would you increase community safety after the pandemic?
11. What are the most critical steps to take after witnessing a post-pandemic sexual assault or rape, regardless of gender?

# 12. What actions should someone in your community take to seek justice for violence or gender-based violence after the pandemic? 13. What challenges may arise when seeking justice for post-pandemic gender-based violence cases? 14. How can we avoid gender-based violence in the community following COVID-19?

# Appendix 2: Key Informant Interview Guide 1

**Key informants Interview Guide 1 (Community opinion leaders, traditional leaders)**

My name is Everjoy Magwegwe, a Post Doctoral Research Fellow with the South Africa research chair in South African Arts and Visual Culture. I would like to thank you very much for agreeing to participate in the interview. The purpose of the interview is to understand your their experiences and perspectives regarding gender-based violence during the COVID-19 pandemic in Golden Valley. I assure you privacy and confidentiality to whatever is going to be discussed in this conversation, no names are to be used throughout this interview.

Name of interviewer : .................................................................

Age : .................................................................

Sex : ..................................................................

Pseudonym : ..............................................................

Date : ................................................................

Questions

1. How do you characterize gender-based violence in Golden Valley?
   2. How do men and women differ in terms of economic independence, family decision-making, and marriage in this community?
   3. How prevalent do you believe GBV is in this population, both before and after the pandemic?
   4. Which groups or persons in this community are disproportionately harmed by gender-based violence?
   5. What are the key causes of gender-based violence in Golden Valley?
   6. Which gender is more likely to conduct violence in this community?
   7. Are there any social stigmas or beliefs that may deter victims from reporting GBV?
2. What additional variables, besides stigma, may contribute to undetected GBV in this community?
   9. How do local culture and community values impact attitudes towards GBV, particularly toward women?
   10. Are there any support services available for victims of gender-based abuse in Golden Valley?
   11. What problems do survivors of GBV have when seeking assistance, especially during and after the pandemic?
   12. What efforts should be implemented to eliminate GBV in this community?
   13. What should Golden Valley's community plan include to combat sexual assault and other types of gender-based violence?

# Appendix 3: Key Informant Interview Guide 2

**Key informants Interview Guide 2 (Health personal and police personal)**

My name is Everjoy Magwegwe, a Post Doctoral Research Fellow with the South Africa research chair in South African Arts and Visual Culture. I would like to thank you very much for agreeing to participate in the interview. the purpose of the interview is to understand your their experiences and perspectives regarding gender-based violence during the COVID-19 pandemic in Golden Valley. I assure you privacy and confidentiality to whatever is going to be discussed in this conversation, no names are to be used throughout this interview.

Name of interviewer : .................................................................

Age : .................................................................

Sex : ..................................................................

Pseudonym : ..............................................................

Date : ................................................................

1. What are gender differences in education job prospects, and financial independence?
2. What is the community's perspective of gender-based violence?
3. Are the victims seen as responsible?
4. Are there therapeutic options available for various types of gender-based violence?
5. Are healthcare facilities adequately prepared and manned with skilled personnel?
6. Do healthcare professionals actively check for GBV? How?
7. Do community health professionals conduct outreach to the community?
8. How many GBV cases are reported annually?
9. How many culprits have been tried, convicted, and sentenced?
10. What community laws and policies address gender-based violence?

11. Do women participate in official and informal group leadership structures?
12. Do the delegates and decision-makers act fairly?
13. Are there any formal or informal women's communities or cooperatives?
14. Who else should we discuss about GBV in this community?

# Appendix 4: Observation Guide

**OBSERVATION GUIDE**

1. Community-related behaviors

- Observe how men and women adjusted their everyday routines and responsibilities during the epidemic.
- How has COVID-19 affected gender relations and responsibilities?

2. Attitudes of Men and Women Towards Each Other

- Determine if the epidemic has impacted mutual respect or increased conflicts between genders.
- Is there evidence of greater conflict, collaboration, or reliance in the post- COVID environment?

3. Analyze nonverbal communication between men and women in the community to identify signs of fear, frustration, or resilience after COVID-19.

- Are there any signs of economic or health-related worry in gestures or communication?

1. Police records of community violence, including gender-based violence.
   - Examine post-COVID police data for any increase or decrease in GBV incidences.
   - Have there been any changes in the types or frequency of violence recorded since the pandemic began?

   5. Health records from the Community Clinic:

- Determine if the number of GBV-related health cases grew or reduced following the pandemic.
- Look for trends that connect COVID-19 to stress-related or economically driven GBV cases.

6. Living Conditions and Livelihood:

- Evaluate the impact of COVID-19 on men and women's living standards, resource access, and economic stability.
- Has the pandemic increased or decreased the community's economic and social vulnerability?

7. Social Interactions of Community Members

- Examine how COVID-19 has affected social gatherings and interactions, particularly between men and women.
- Have there been any visible changes in social engagement or public discussion of GBV-related issues?

8. Infrastructure Development:

- Evaluate the impact of the pandemic on critical services and infrastructure, including healthcare, police, and community centers.
- Are there any new resources available to address the impact of GBV following COVID-19?
